# 4203 Re-engineering the Approach to Extremely Preterm Breech Deliveries with Student Led Team Science

**DOI:** 10.1017/cts.2020.358

**Published:** 2020-07-29

**Authors:** Alissa Dangel, Michael House, Kumaran Kolandaivelu, Gordon Huggins, Nevan Hanumara, Alexander Slocum

**Affiliations:** 1 Tufts University; 2 Division of Maternal Fetal Medicine, Tufts University; 3 Massachusetts Institute of Technology

## Abstract

OBJECTIVES/GOALS: Vaginal delivery is typically avoided in extremely preterm breech fetuses due to the concern for head entrapment by the cervix. Development of a device to prevent head entrapment would be best addressed by a multidisciplinary approach incorporating engineering principles with clinical obstetrics. METHODS/STUDY POPULATION: Construction of a collaborative multidisciplinary team to address the clinical challenge of preventing head entrapment was initiated through a unique course at the Massachusetts Institute of Technology (Course 2.75, Medical Device Design). The course would provide a structured means by which students (senior undergraduate and graduate students in Mechanical Engineering) would be paired with a clinical advisor and faculty in their department. Weekly team meetings were scheduled to review the clinical context pertinent to the problem and review engineering principles needed to develop a solution. The course also provided a small monetary budget ($4K) for the students to purchase supplies. RESULTS/ANTICIPATED RESULTS: During the semester long course, several iterations of a prototype were designed. Each subsequent rendition was evaluated from both an engineering and manufacturing perspective, as well as clinical appropriateness. The weekly meetings allowed for rapid re-design and assured that all necessary parameters were considered by the entire team. Students also had access to lab facilities and additional mentorship that allowed for supplementary input beyond that generated by core team members. These interactions, along with those of their classmates working on other projects, provided a strong base for exploring subsequent device development. DISCUSSION/SIGNIFICANCE OF IMPACT: Successful medical device development requires a collaborative process and students can be ideal members of these teams as they reside in an environment that is conducive to exploration and novel idea generation. Course-based student led team science platforms can provide an excellent foundation for solving uniquely challenging medical problems.

